# Factors Influencing Information Service Quality of China Hospital: The Case Study of since 2017 of a Hospital Information Platform in China

**DOI:** 10.1155/2020/2089024

**Published:** 2020-07-01

**Authors:** Lei Jiao, HuaPing Xiao, XiaoZhuo Zhu, Xu Zhao

**Affiliations:** ^1^Yale School of Medicine, Yale University, New Haven, CT 06510, USA; ^2^Department of Anesthesiology, Jiangxi Cancer Hospital, Nanchang, Jiang Xi 330029, China; ^3^College of Public Service and Management, Ningbo College of Health Sciences, Ningbo, Zhejiang 315100, China; ^4^Department of Anesthesiology, The Second Xiangya Hospital, Central South University, Changsha, Hunan 410011, China

## Abstract

*Background*: As a country with the largest number of netizens around the world, China enjoys improving social information services based on the Internet. With such a large quantity of network users, it is inevitable for China's hospitals at various levels to provide patients and the public with information services by setting up their own official websites. But it is still elusive for the factors affecting the information service quality of China Hospital. *Objective*: Identifies the factors affecting the information service quality of the case of the Online website of a hospital in China.adding new content to the research fruits in this field. The research can effectively enhance the efficiency of hospital resource utilization, allocating limited resources to most efficient areas and leveling up the information service quality of hospitals to the largest extent. This ultimately improves patient satisfaction. *Method*: This research investigates the factors affecting the information service quality of the case of a Chinese hospital online website and by means of Delphi method, statistical analysis, and other research methods, formulates the Evaluation Indicator System for the Information Service Quality of the case of the Online website of a hospital in China. The research applies this system to the empirical research on the information service quality of the hospital's website and then makes a comparative analysis between the research results and traffic data of the websites of other hospitals over the same period. *Results*: By means of the Bivariate Correlation, the author carried out a correlation analysis of the comprehensive evaluations of the information service of the Online website of a hospital in China and the traffic data of the Online website of a hospital in China, including the total traffic, PV and UV. For details of the analysis results, indicates that the correlation coefficient among the three objects is 1, a significant correlation. It also suggests that the comprehensive evaluations of the information service of A Chinese hospital website and the traffic of A Chinese hospital website are positively correlated. The information service quality of China Hospital website is an important component of the hospital's overall service quality. This research on the information service quality of China Hospital website covers the website's service functions, service quality, resources and the front-end and back-end technology systems. *Discussion and conclusion*: In the case that the China Hospital information service function is still not perfect, perfecting the functions of China Hospital website plays a decisive role in improving the information service quality of the hospital. In addition, it can be inferred that after the information service function of China Hospital website is improved or the evaluation of the functional quality attribute of website information service scores higher, the supporting attribute of website information service will be the next key task for the hospital in enhancing its information service quality is the improvement ratio of Functional quality attribute of website information service and the supporting attribute of website information service tend to be the same., And even the improvement rate of the supporting attribute is sometimes higher than the improvement rate of Functional quality. So The construction of a model of the comprehensive evaluation system on the information service of has pointed out a new direction in China's research in this field, This model is both of high theoretical value and practical value.

## 1. Introduction

According to statistics of China Internet Network Information Center (CNNIC), the number of Chinese netizens, including Internet users on mobile phones, has reached 1.701 billion, which is the largest number in the world, and this number has been increasing year by years [1]. Therefore, the Internet has already been the main approach for the public to acquire information in China. In the meanwhile, medical institutions that provide services for the public are also carrying through the informatization reform. The hospital information platforms thus become the main representative in this reform. Hospitals usually provide more than just medical treatments to patients. They also offer other services including information service. Among them, using hospital information platform is one of the important approaches for a hospital to provide information service to patients. After ten years of informatization, as of 2019, the average rate of third-level hospitals launching information platforms in each province of China has reached 82% [2]. China Hospitals usually provide more than just medical treatments to patients. They also offer other services including information service, consultation service, and psychological service. Among them, using hospital information platform is one of the important approaches for a hospital to provide information service to patients.

In China's mainland, when patients have medical needs and choose hospitals online, they tend to choose a hospital that has high quality information service on the hospital website. They gain information service on the website and proceed with subsequent medical service accordingly. They usually recommend the website providing good experience of online information service to their families or friends in need of medical service. It can be seen that an advanced hospital information platform with high quality information service can not only bring profits to the hospital but also establish the hospital reputation, and more importantly patients can enjoy high quality information service. Therefore, it is of importance to study factors influencing information service quality of hospital information platforms so as to improve their information service quality.

Research on information service quality of hospital information platforms is firstly carried out on information service quality of hospital information system, such as the measurement of effectiveness of the information system and the study on SERVQUAL scale that is used to measure the service quality of information system [3, 4]. Then, with the popularity of computers and networks as well as the increase in web browsing, the academic community pays more attention to the research on the service quality of the information system of webpages since 2000. Tan in 2003 studied the service quality frame of the information system [5]. Pinho in 2011 studied the influences of network service quality on network usage [6]. Baldwin in 2014 studied the relationship between the rating of patients' satisfaction and the quality of hospital information platforms and measures [7]. However, China's research on information service quality of hospital information platforms has just started. The major contributions have been made mainly by some scholars in Taiwan and coastal universities in China's mainland. For example, Liang in 2009 conducted a research on the influences of the quality of information platforms on the performance of an organization's relationship with customerss [8]. Pei and Wang in 2009 researched on the evaluation system of information service quality of user-oriented information platforms [9]. Zhu and Xiong in 2012 studied the evaluation criteria on information service quality of medical information platforms [10]. This article shows investigating and studying the information platform of a Hospital Information Platform from the perspective of management, the thesis intends to identify factors influencing the information service quality of hospital information platforms, so as to figure out factors affecting the information service quality of the information platform of a Hospital Information Platform in China and the interaction among these factors.

## 2. Methods

This study aimed to understand Factors Influencing Information Service Quality of the Information Platform of China Hospital. To achieve this goal a case study and Delphi and Questionnaire methodology were followed. This study will discuss information service quality of hospital information platforms in two aspects—the essence of the information service of hospital information platforms and perception of information service. Then an evaluation system will be established and verified.

To begin with, following the case study method, this study's object is set within a certain range in order to perform in-depth and comprehensive analysis through dynamic follow-up investigation into them. This study selects the a Hospital Information Platform in China as the object of the case study. Through study design, data collection, data analysis, and study report draft, factors influencing information are sought out as reference for the improvement of service quality of hospital information platforms in China.

Second, Use Delphi and Questionnaire method. In this study Use Delphi, experts in this field are consulted to discuss how to determine or quantify indicators of various dimensions, indicator weights of medical information service quality of information platforms in Chinese hospitals so as to help finish the study [11, 12]. In this study Use Questionnaire, In this Questionnaire method, the subjects are given brief forms and asked to offer suggestions or file complaints on certain issues so as to collect information. Based on literature research nd experts' views, a questionnaire is designed for the front-line hospital staff and patients. The questionnaire consists of hospital staff investigation, patient investigation and expert investigation. Every investigation will be carried out two times, and there are two rounds for all the investigations. The first investigation is on the dimension structure of evaluation system for medical information service function and quality of a Hospital Information Platform in China, and the second one is on evaluation system index coefficient of medical information service function and quality of a Hospital Information Platform in China. At last, Statistical Analysis. In this study, Excel is used for data-entry of information in the expert consultation, and SPSS is used for statistical analysis of data in the questionnaire so as to examine the consistency and logic of the data and to ensure the correctness of the data.

## 3. Comprehensive Evaluation System for Medical Information Service of a Hospital Information Platform in China

### 3.1. The Structure of System Evaluating the Information Service

**Figure 1:**
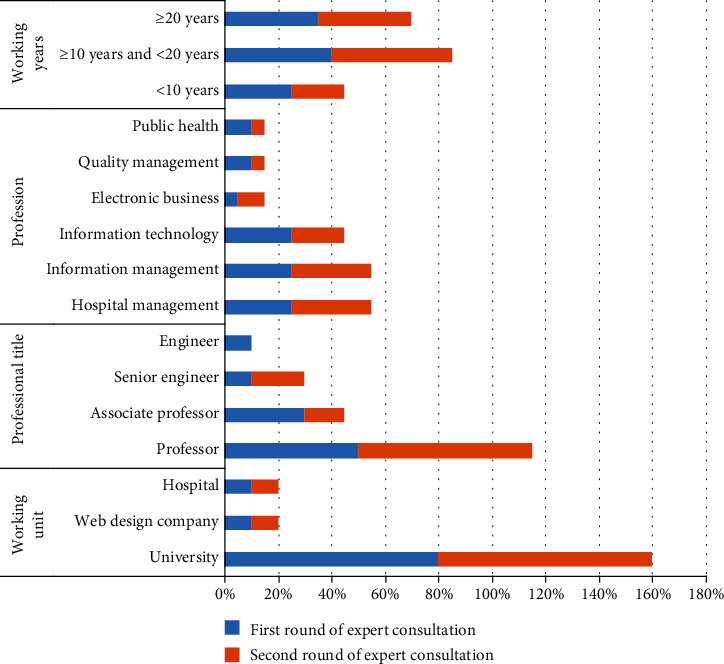
Statistics of Status of Consultation Experts.

This study has randomly chosen 101 experts across China to put into the expert bank of consultation object, ranging from professors in management or other disciplines with deputy senior or above titles to information technology experts at hospitals and professional technicians at information technology web design companies. Next, send the initial dimension indicator set to about 20 experts via mobile phones, letters and emails for at least two rounds of consultation to settle the names, categories, quantity and structure of the evaluation system's dimension indicators. Each round of consultation randomly chooses experts among the 101 consultation objects to send consultation forms, See Figure 1 for details.

The expert difference coefficient Q indicates the difference of expert opinion. This variable coefficient demonstrates different degree of expert recognition of the evaluated indicators. The smaller the variable coefficient is, the less the difference of expert opinion is. Calculation equation of variable coefficient:

(1) $$\begin{array}{c} Q_{i}=\frac{s_{i}}{v_{i}} \end{array}$$

In the equation, Qi refers to the variable coefficient of indicator i, si standard deviation and vi weighted arithmetic mean. In the two rounds of expert consultations, 45 consultation forms were sent out, and 40 valid feedbacks were collected. The effectiveness of the two rounds of consultations was 86%, both of which were higher than 60%.18 dimensional indicators' difference coefficients in the first round are over 0.25, and all these 18 dimensions have been adjusted accordingly; while the difference coefficients of 73 dimensional indicators are all between 0.08-0.24 (less than 0.25). In the second round, only one dimension's difference coefficient is bigger than 0.25, which has been modified, and the difference coefficients of 72 dimensional indicators are all between 0-0.24 (less than 0.25).

**Figure 2:**
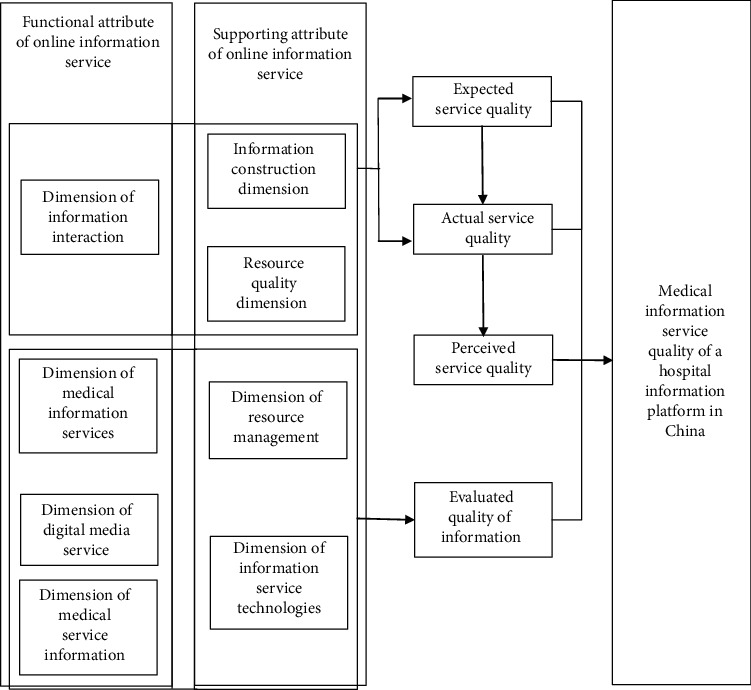
Theoretical Research Platform of Information Service Quality of a Hospital Information Platform in China (Formal Version).

**Figure 3:**
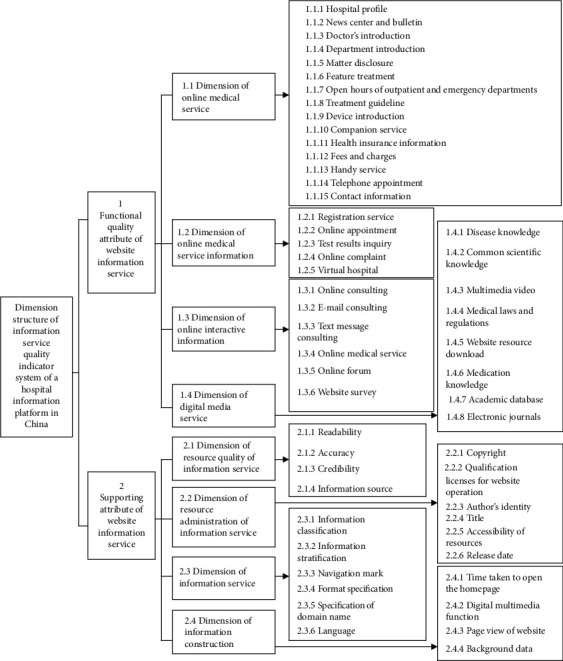
Dimension Structure of Information Service Quality Indicator System of a Hospital Information Platform in China (Final Version).

In summary, The expert authority degree is 0.846. In the Delphi Method, when the expert authority degree >0.7 it can be seen as relatively high. Hence, the expert authority degree in this round of expert consultation is quite good. Experts have given positive responses to the dimension structure of the indicator system in this thesis quite unanimously, which demonstrates that both the first and second round of research have been recognized by consultation experts as scientific and rational. The attributes, dimensions and indicators of the information service evaluation system of a Hospital Information Platform in China have been identified. According to Their respective arithmetic averages are less than 3, which does not reach the predetermined indicator of consistency. After two rounds of questionnaires, the old indicators are eliminated to construct new indicators. Based on the above indicators, this thesis has developed it into Figures 2 and 3 the formal version.

### 3.2. System Construction of Information Service Quality Indicators and Weight Determination

Through the Delphi method, this thesis conducts two rounds of expert consultation and discussion according to the importance of the dimensional indicators of information service evaluation system of a Hospital Information Platform in China. The consultation form is basically the same as the one constructed in the information service evaluation system in the hospital information platform. The difference lies in that the consulting content is changed into the 4 initial weight schemes and the experts can either choose a scheme from the above four schemes or propose their own ones. If the experts do not have a unified opinion after the first round of consultation, the author can merge and sort out the four schemes and the schemes put forward by experts in the first round.

Through the first and second rounds of expert consultation, the final weight distribution scheme is determined. The combined weight values of secondary and tertiary indicators are obtained through multiplication. The combined weight values of the secondary indicators are obtained by Equation (2), while those of tertiary indicators are got by Equation (3).

(2) $$\begin{array}{c} S_{wi}=S_{1i}\times S_{2i} \end{array}$$

*S_wi_* is the combined weight value of the secondary indicators, S_1*i*_ refers to the weight of primary indicator corresponding to indicator *i*, and S_2*i*_ the weight of secondary indicator corresponding to indicator *i*. See Table 1.

(3) $$\begin{array}{c} S_{wi}=S_{1i}\times S_{2i}\times S_{3i} \end{array}$$

*S_wi_* is the combined weight value of the tertiary indicators, *S_1i_* refers to the weight of primary indicator corresponding to indicator *i*, *S_2i_* the weight of secondary indicator corresponding to indicator *i*, and *S_3i_* the weight of tertiary indicator corresponding to indicator *i*. The weighting allocation interval is [0,1], See Table 1.

### 3.3. Explanation of Evaluation Indicator Assignment

After determining the structure and weight of the comprehensive evaluation system, to improve it and strengthen its operability, there is still need to take different quantitative assignment methods in accordance with the characteristics of each indicator. Since the assignment interval of all the indicators in the comprehensive evaluation system is set within [0, 1]. According to the characteristics of each indicator, different grades and quantified assignments are divided, and the quantitative scores of all the indicators are calculated. For details about the quantitative assignment of all standards in the comprehensive evaluation system, see Table 2.

### 3.4. Indicator Quantification of Evaluation System and Assignment Standard and Construction of Comprehensive Evaluation Model

By weighting the dimensions of the system and assigning values in a quantitative way, the author establishes a comprehensive evaluation model for the information service of the a Hospital Information Platform in China according to the weight of its information service and the quantitative value assignment standards. For the total weighted score of the comprehensive indicators, see Equation (4).

(4) $$\begin{array}{c} R=\overset{m}{\underset{i=1}{\sum }}\overset{n_{i}}{\underset{j=1}{\sum }}W_{ij}R_{ij}\times 100 \end{array}$$

R representing the total score is in the range between 0 and 100, Rij is the value of the j indicator in the i dimension, and Wij is the combined weight of the j indicator in the i dimension. i is 1 to m, j is 1 to ni, ni is the number of indicators covered by the i dimension, m is the number of the dimensions and equals 8, and S is the number of indicators in the tertiary level and equals 54.

The author first collects data by using the Hospital Website Information Service Evaluation Form, then processes the data, assigns values in a quantitative way according to the evaluation of indicators at each level, and in the end, uses the equation of the comprehensive evaluation model to calculate the comprehensive score of the information service of the a Hospital Information Platform in China.

## 4. Empirical Research Results

Since the website of a Hospital Information Platform in China is the only channel for the hospital's information platform to deliver information services, the author sets the website of a Hospital Information Platform in China as the object of the empirical research. Established on June 19, 2013, the website of a Hospital Information Platform is redesigning its overall pages on June 19, 2017, as the empirical research goes on. Therefore, the author sets both the pre-revised website and the post-revised website as the object of the empirical research. Formulated on the basis of the Comprehensive Evaluation Model for the Information Service of a Hospital Information Platform Information Platform, the a Hospital Information Platform Website Information Service Evaluation Form is an online data collection form for the evaluation of hospitals' website information service.

In the empirical research, December, 2016 to January, 2020, 80 participants falling of Internet Explorer 11 as the uniform browser and logged in a Hospital Information Platform's website in ten periods. Stratified random sampling is adopted to select participants, and in accordance with the sampling indicators, these participants are classified into four groups: out-patients, in -patients, hospital employees, and the public and professionals.TThrough the empirical research, we get the comprehensive evaluations of the information service of a Hospital Information Platform's information platform. if participants give different scores for evaluations of these two items, a mean value is adopted as the final score. The total averages of Pre-revision are: 19.4 and 20.3875, and the total averages of Post-revision are: 73.5, 74.4, 77.7, 77.3, 77.5, 79.4, 81.7, 84.3.Data in show that after the overall revision, the website of a Hospital Information Platform gets clearly higher comprehensive evaluations for its information service. (for details, see Table 3).

By means of the Bivariate Correlation in SPSS, the author carried out a correlation analysis of the comprehensive evaluations of the information service of a Hospital Information Platform's information platform and the traffic data of a Hospital Information Platform website, including the total traffic, PV (page view) and UV (unique visitor). The total traffic is equal to PV plus UV. For details of the analysis results, the correlation coefficient among the three objects is 1, a significant correlation. It also suggests that the comprehensive evaluations of the information service of a Hospital Information Platform's information platform and the traffic of a Hospital Information Platform's website are positively correlated. That is to say, the traffic of a Hospital Information Platform's website will vary to the changes in the comprehensive evaluations of the information service of a Hospital Information Platform's information platform.

## 5. Conclusions

**Figure 4:**
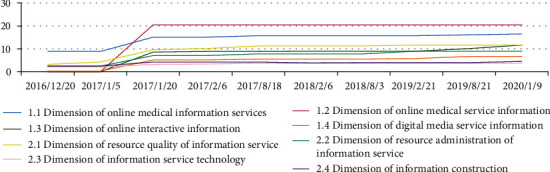
Line Chart of the Correlation Analysis of the Comprehensive Evaluations of The Secondary Indicators of The Information Service of a Hospital in China.

Based on the actual situation of Chinese hospitals, creates a theoretical research platform of medical information service quality based on the reality of a Hospital Information Platform, and sorts out the core of website information service of Chinese hospitals as well as the influencing factors and their correlations. Focusing on the function and quality of hospital information service, it has established a complete and scientific evaluation system of hospital information service that conforms to the actual situation of Chinese hospitals. Still, through the empirical research on a Hospital Information Platform website, it also validates the scientificity, rationality and practicability of the model proposed in this thesis. The model's scores in various dimensions in empirical researchSee Figure 4 for details.

**Figure 5:**
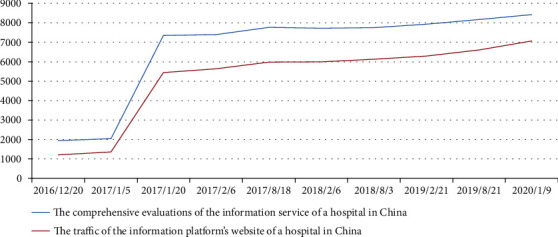
Line Chart of the Correlation Analysis of the Comprehensive Evaluations of the Information Service of a Hospital in China and the Traffic of Information Platform's Website of a Hospital in China (Comprehensive Evaluations of the Information Service of a Hospital in China of value = value×100).

For the first time in China, the author conducts comparison and correlation analysis of survey results on comprehensive evaluation of hospital website information service and traffic data through empirical research, in order to discuss the relations between the evaluation results and the traffic data. And through the above empirical research to prove the positive correlation between the two, see Figure 5 for details.

## 6. Suggestions

The results of the empirical research show Enriching the information service functions plays an important role for hospitals to improve the information service quality of their websites and their website usage. The weight of the functional quality attribute of website information service is 0.7, significantly higher than that of the supporting attribute. In addition, the second-level indicators of the functional quality attribute are the dimensional indicators for measuring the functions of hospital websites, while in the evaluation on January 9, 2020, the evaluation score of the functional quality attribute is 55.41, suggesting a improving.

**Figure 6:**
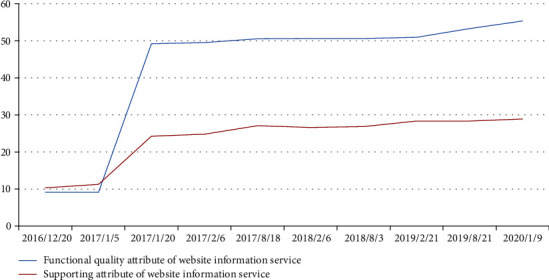
Line Chart of the Functional quality attribute of website information service and Supporting attribute of website information service.

As the evaluation of the functional quality attribute of website information service shows a large room for improvement, perfecting the information service function of a Hospital Information Platform's website is the key task that the hospital should do to enhance its information service. In addition, that after the information service function of a Hospital Information Platform's website is improved or the evaluation of the functional quality attribute of website information service scores higher, the supporting attribute of website information service will be the next key task for the hospital in enhancing its information service quality. From the data in Figure 6 and Table 3, we can see that the improvement ratio of Functional quality attribute of website information service and the supporting attribute of website information service between 2017/8/18 and 2020/1/9 tend to be the same. And even the improvement rate of the supporting attribute is sometimes higher than the improvement rate of Functional quality.

All in all, between 2017/8/18 and 2020/1/9, the final score of the functional quality attribute of the website information service in 2020/1/9 is about 6 times that of 2017/8/18, and the support attribute of the website information service The final score in 2020/1/9 is about 3 times that of 2017/8/18,see Figure 6 and Table 4 for details.

This study is the first one in China that applies both the Delphi method and composite grade method to construct the model of comprehensive evaluation system of hospital information service. For the first time in China, the author conducts comparison and correlation analysis of survey results on comprehensive evaluation of hospital website information service and traffic data through empirical research, in order to discuss the relations between the evaluation results and the traffic data. Therefore, it bears considerable reference value. This model is both of high theoretical value and practical value. But the development of new network information technology often goes beyond hospital managers imagination. Therefore, the information service evaluation system in this thesis will be enriched with the development of the times and information technology.

## Figures and Tables

**Table 1:** Indicator Combined Weight Values of Information Service Evaluation System of a Hospital Information Platform in China.

(1) distribution scheme of primary indicators			
No.	Evaluation Indicator	Scheme 4	Weight value
1	Functional quality attribute of website information service	70	0.7
2	Supporting attribute of website information service	30	0.3
Total			1
(2) distribution scheme of secondary indicators			
No.	Evaluation Indicator	Scheme 3 Original first-round my scheme 2)	Combined weight value
1.1	Dimension of online medical information services	35	0.245
1.2	Dimension of online medical service information	30	0.21
1.3	Dimension of online interactive information	20	0.14
1.4	Dimension of digital media service information	15	0.105
No.	Evaluation Indicator	Scheme 2	Combined weight value
2.1	Dimension of resource quality of information service	40	0.12
2.2	Dimension of resource administration of information service	30	0.09
2.3	Dimension of information service technology	15	0.045
2.4	Dimension of information construction	15	0.045
Total			1
(3) distribution scheme of tertiary indicators			
No.	Evaluation Indicator	Scheme 1	Combined weight value
1.1.1	Hospital profile	10	0.0245
1.1.2	News and bulletin	7	0.01715
1.1.3	Introduction of hospital experts	7	0.01715
1.1.4	Introduction of departments	5	0.01225
1.1.5	Publicity of medical events	5	0.01225
1.1.6	Special medical treatment	5	0.01225
1.1.7	Emergency service and outpatient service hour	10	0.0245
1.1.8	Treatment guidelines	10	0.0245
1.1.9	Device introduction	10	0.0245
1.1.10	Accompanying services	4	0.0098
1.1.11	Medical insurance information	5	0.01225
1.1.12	Charging standard notes	5	0.01225
1.1.13	Handy Services	5	0.01225
1.1.14	Appointments and registration by phone	6	0.0147
1.1.15	Contact information	6	0.0147
No.	Evaluation Indicator	Scheme 4	Combined weight value
1.2.1	Signing in service	25	0.0525
1.2.2	Online appointment and registering	35	0.0735
1.2.3	Examination results query	25	0.0525
1.2.4	Online complaint	10	0.021
1.2.5	Virtual hospital	5	0.0105
No.	Evaluation Indicator	Scheme 4	Combined weight value
1.3.1	Online advisory	30	0.042
1.3.2	E-mail advisory	10	0.014
1.3.3	Text message advisory	10	0.014
1.3.4	Online medical service	30	0.042
1.3.5	BBS	15	0.021
1.3.6	Online message board	5	0.007
No.	Evaluation Indicator	Scheme 2	Combined weight value
1.4.1	Disease knowledge	15	0.01575
1.4.2	Popular science	15	0.01575
1.4.3	Multimedia video	7.5	0.007875
1.4.4	Medical policy	15	0.01575
1.4.5	Medical education	7.5	0.007875
1.4.6	Drug usage	15	0.01575
1.4.7	Academic database	12.5	0.013125
1.4.8	Hospital E-journal	12.5	0.013125
No.	Evaluation Indicator	Scheme 2	Combined weight value
2.1.1	Readability	20	0.024
2.1.2	Accuracy	30	0.036
2.1.3	Completeness	20	0.024
2.1.4	Information source	30	0.036
No.	Evaluation Indicator	Scheme 4	Combined weight value
2.2.1	Copyright	35	0.0315
2.2.2	Qualification licenses for website operation	25	0.0225
2.2.3	Author's identity	10	0.009
2.2.4	Title marking	10	0.009
2.2.5	Accessibility of resource	10	0.009
2.2.6	Release date	10	0.009
No.	Evaluation Indicator	Scheme 3	Combined weight value
2.3.1	Information classification	25	0.01125
2.3.2	Information stratification	10	0.0045
2.3.3	Navigation mark	20	0.009
2.3.4	Format specification	20	0.009
2.3.5	Specification of domain name	10	0.0045
2.3.6	Language	15	0.00675
No.	Evaluation Indicator	Scheme 4	Combined weight value
2.4.1	Time taken to open the homepage	30	0.0135
2.4.2	Digital multimedia function	20	0.009
2.4.3	Website page view	20	0.009
2.4.4	Background data	30	0.0135
Total			1

**Table 2:** The Quantification and Assignment Standards of the Dimensions of Online Medical Information Services.

No.	Evaluation standards	Description of quantitative assignment
Quantitative assignment of the dimensions of online medical information services		
1.1.1	Hospital profile	The hospital profile item includes ten sub-items: (introduction, logo, history, leadership, culture, qualification, honor, environment, words by the president and video of the hospital.) each of these items has two levels: “Established” valued 1 and “to be established” valued 0. The calculation of the value of the quantitative standard is: 1/*n*∑_*i*=1_^*n*^*W*_*i*_ “*n*” is the number of the sub-items and equals 10. *W_1_* is the specific value of the *i* sub-item.
1.1.2	News and bulletin	The news and announcement include two sub-items: News and announcement, respectively, valued 0.7 and 0.3. According to the updating frequency, the sub-item news falls into four categories, no updating, updating every one month and over, updating every week and updating every two days, which are valued, respectively, 0, 0.34, 0.67 and 1. The sub-item announcement has two levels: “Established” valued 1 and “to be established” valued 0. The calculation of the value of the quantitative standard is: 0.7 × *W*_1_ + 0.3 × *W*_2_ *W_1_*is the value of news and *W_2_* is that of announcement.
1.1.3	Introduction of hospital experts	The introduction of hospital experts includes five sub-items: (personal introduction, expertise, honor and academic achievements, positions and contacts.) this item has two levels: “Established” valued 1 and “to be established” valued 0. The calculation of the value of the quantitative standard is: 1/*n*∑_*i*=1_^*n*^*W*_*i*_ “*n*” is the number of the sub-items and equals 5. *W_i_* is the specific value of the *i* sub-item.
1.1.4	Introduction of departments	The item introduction of departments includes five sub-items: (departments introduction, expertise, medical teams, honor and contacts.) it has two levels: “Established” valued 1 and “to be established” valued 0. The calculation of the value of the quantitative standard is: 1/*n*∑_*i*=1_^*n*^*W*_*i*_ “*n*” is the number of the sub-items and equals 5. *W_i_* is the specific value of the *i* sub-item.
1.1.5	Publicity of medical events	The publicity of medical events includes five sub-items: (clinical service management, nursing management, hospital regulations, academic research and medical education.) it has two levels: “Established” valued 1 and “to be established” valued 0. The calculation of the value of the quantitative standard is: 1/*n*∑_*i*=1_^*n*^*W*_*i*_ “*n*” is the number of the sub-items and equals 5. *W_i_* is the specific value of the *i* sub-item.
1.1.6	Special medical treatment	The special medical treatment includes three sub-items: Programs, introduction and effects. It has two levels: “Established” valued 1 and “to be established” valued 0. The calculation of the value of the quantitative standard is: 1/*n*∑_*i*=1_^*n*^*W*_*i*_ “*n*” is the number of the sub-items and equals 3. *W_i_* is the specific value of the *i* sub-item.
1.1.7	Emergency service and outpatient service hour	The emergency service and outpatient service hour include two sub-items: Time and doctors. It has two levels: “Established” valued 1 and “to be established” valued 0. The calculation of the value of the quantitative standard is: 1/*n*∑_*i*=1_^*n*^*W*_*i*_ “*n*” is the number of the sub-items and equals 2. *W_i_* is the specific value of the *i* sub-item.
1.1.8	Treatment guidelines	The treatment guidelines include eight sub-items: (transportation guides, hospital indoor navigation, outpatient information, registration information, emergency information, medical test information, inpatient treatment and discharge guidelines.) it has two levels: “Established” valued 1 and “to be established” valued 0. The calculation of the value of the quantitative standard is: 1/*n*∑_*i*=1_^*n*^*W*_*i*_ “*n*” is the number of the sub-items and equals 8. *W_i_* is the specific value of the *i* sub-item.
1.1.9	Device introduction	The device introduction includes five sub-items: (types, names, functions, distribution and numbers.) it has two levels: “Established” valued 1 and “to be established” valued 0. The calculation of the value of the quantitative standard is: 1/*n*∑_*i*=1_^*n*^*W*_*i*_ “*n*” is the number of the sub-items and equals 5. *W_i_* is the specific value of the *i* sub-item.
1.1.10	Accompanying service	The accompanying service includes five sub-items: (home care, inpatient care, postpartum care, pregnancy care and others.) it has two levels: “Established” valued 1 and “to be established” valued 0. The calculation of the value of the quantitative standard is: 1/*n*∑_*i*=1_^*n*^*W*_*i*_ “*n*” is the number of the sub-items and equals 5. *W_i_* is the specific value of the *i* sub-item.
1.1.11	Medical insurance information	The medical insurance information includes four sub-items: (news, policies, guidelines, and organizations.) it has two levels: “Established” valued 1 and “to be established” valued 0. The calculation of the value of the quantitative standard is: 1/*n*∑_*i*=1_^*n*^*W*_*i*_ “*n*” is the number of the sub-items and equals 4. *W_i_* is the specific value of the *i* sub-item.
1.1.12	Charging standard notes	The charging standard notes include six sub-items: (outpatient, emergency and comprehensive medical services, traditional Chinese and folk medicine, medical technologies, clinical treatment, special medical treatment services and pharmaceutical drugs.) it has two levels: “Established” valued 1 and “to be established” valued 0. The calculation of the value of the quantitative standard is: 1/*n*∑_*i*=1_^*n*^*W*_*i*_ “*n*” is the number of the sub-items and equals 6. *W_i_* is the specific value of the *i* sub-item.
1.1.13	Handy Service	The item Handy Services includes ten sub-items: (guiding services, drinking water, body temperature measurement, Presbyopic glasses, wheelchairs and stretchers, medical record copying, free magazines, parking guides, testing report post service and others.) it has two levels: “Established” valued 1 and “to be established” valued 0. The calculation of the value of the quantitative standard is: 1/*n*∑_*i*=1_^*n*^*W*_*i*_ “*n*” is the number of the sub-items and equals 10. *W_i_* is the specific value of the *i* sub-item.
1.1.14	Appointments and registration by phone	According to its functions, the item appointments and registration by phone has four levels: (1) no telephone appointment service is provided on the website, which is valued 0 (2) the website offers telephone number for appointment but it is not accessible. The value is 0.34 (3) the website has accessible telephone number but users cannot make appointment through it. The value is 0.67 (4) the website provides telephone appointment service and users can make an appointment successfully, which is valued 1 The state that the website provides telephone appointment service and users can make an appointment successfully is: When researchers acting as patients make an appointment over the telephone, operators answer the phone promptly, make a registration in appropriate departments for the patients according to the symptoms described by the researchers, record the patients' information and thus finish all the registration procedures.
1.1.15	Contact information	The contact information includes ten sub-items for contacting the hospitals: (hospital switchboard, phone numbers of departments, discipline inspection phone number, E-mail address, hospital address, location, traffic, digital map, President's mail-box and others.) it has two levels: “Established” valued 1 and “to be established” valued 0. The calculation of the value of the quantitative standard is: 1/*n*∑_*i*=1_^*n*^*W*_*i*_ “*n*” is the number of the sub-items and equals 10. *W_i_* is the specific value of the *i* sub-item.
Online medical service information		
1.2.1	Signing in service	According to its functions, the signing in service has four levels: (1) the hospital website does not provide registration service. The value is 0 (2) the website provides registration service but users cannot finish the registration. The value is 0.34 (3) the website has registration service and users can register successfully. The value is 0.67 (4) the website has registration service and users can register successfully and use the registered accounts to use website's functions. The value is 1
1.2.2	Online appointments and registering	The online appointments and registering has two levels: “Established” valued 1 and “to be established” valued 0.
1.2.3	Examination results query	According to patients' names and medical service numbers, the examination results query has two levels: “Established” valued 1 and “to be established” valued 0.
1.2.4	Online complaint	The online complaint item has two levels: “Established” valued 1 and “to be established” valued 0.
1.2.5	Virtual hospital	The virtual hospital includes six sub-items: (medical service appointment, guidelines, specialist clinic, hospital indoor navigation, online test and 3D virtual hospital.) it has two levels: “Established” valued 1 and “to be established” valued 0. The calculation of the value of the quantitative standard is: 1/*n*∑_*i*=1_^*n*^*W*_*i*_ “*n*” is the number of the sub-items and equals 6. *W_i_* is the specific value of the *i* sub-item.
Online interactive information		
1.3.1	Online advisory	Standards of this kind include two parts: Service functions valued 0.6 and service quality valued 0.4. The service functions has two levels: “Established” valued 1 and “to be established” valued 0. In terms of the value of service functions, researchers acting as users send consultation to designated e-mail address or phone number. According to the time period before the consultation is answered and the accuracy of the answers, the service function has four levels: Half a month and over, valued 0, in half a month, valued 0.34, in a week, valued 0.67, and in three days, valued 1. The calculation of the value of the quantitative standard is: 0.6 × *W*_1_ + 0.4 × *W*_2_ W_1_ is the value of the service functions and W_2_ is that of the service quality.
1.3.2	E-mail advisory	
1.3.3	Text message advisory	
1.3.4	Online medical service	According to its functions, the online medical service has three levels: (1) researchers acting as users try the medical services provided on the hospital website to assign the value of the item. If the website does not provide online medical services, the value is 0 (2) if the website provides online medical services but users cannot register and further access these services, the value is 0.5 (3) if the website provides online medical services and these services are accessible for users, the value is 1
1.3.5	BBS	The BBS item has four levels: (1) if the website does not provide the function of online forum, the value is 0 (2) if the website provides this function and users can register and log in to use it, the value is 0.34 (3) if the website has an online forum and users can register, log in and search for information by categories, but the number of posts is relatively small (less than 100), the value is 0.67 (4) if the website has an online forum with various functions, users can register, log in and search for information by categories, and the number of posts is relatively large (more than 100), the value is 1
1.3.6	Online message board	This item has two levels: “Established” valued 1 and “to be established” valued 0.
Digital media service information		
1.4.1	Disease knowledge	According to the amount of its contents, the disease knowledge has four levels: (1) if the website does not provide this function, the value is 0 (2) if the website has this function and the number of knowledge items is no more than 50, the value is 0.34 (3) if the website provides knowledge about diseases and categorizes these knowledge, and the number of knowledge items is between 50 and 100 (100 included), the value is 0.67 (4) if the website provides knowledge about diseases and categorizes these knowledge, and the number of knowledge items is over 100, the value is 1
1.4.2	Popular science	According to the amount of its contents, the popular science has four levels: (1) if the website does not provide this function, the value is 0 (2) if the website has this function and the number of knowledge items is no more than 50, the value is 0.34 (3) if the website provides knowledge about science and categorizes these knowledge, and the number of knowledge items is between 50 (50 included) and 100, the value is 0.67 (4) if the website provides knowledge about science and categorizes these knowledge, and the number of knowledge items is over 100 (100 included), the value is 1
1.4.3	Multimedia video	According to the amount of its contents, the multimedia video has four levels: (1) if the website does not provide this function, the value is 0 (2) if the website has this function and the number of videos is less than 10, the value is 0.34 (3) if the website provides multimedia videos and the number of videos is between 10 (10 included) and 50, the value is 0.67 (4) if the website provides multimedia videos and categorizes videos, and the number of videos is 50 and over, the value is 1
1.4.4	Medical policy	The medical policy has two levels: “Established” valued 1 and “to be established” valued 0.
1.4.5	Medical education	According to the amount of its contents, the medical education has four levels: (1) if the website does not provide this function, the value is 0 (2) if the website has this function and the number of resources for downloading is no more than 10 (10 included), the value is 0.34 (3) if the website provides resources for downloading and the number is over 10, the value is 0.67 (4) if the website provides resources for downloading and categorized these resources, and the number is over 50, the value is 1
1.4.6	Drug usage	The drug usage has two levels: “Established” valued 1 and “to be established” valued 0.
1.4.7	Academic database	The academic database has three levels: An academic database with owned academic resources, an academic database and to be established, which valued, respectively, 1, 0.5 and 0.
1.4.8	Hospital E-journal	According to its function and contents, the hospital E-journal has four levels: (1) if the website does not provide this function, the value is 0 (2) if the website provides this function, and the number of e-journals is no more than 5 (5 included), the value is 0.34 (3) if the website has this function, and the number of e-journals is between 5 and 10 (10 included), the value is 0.67 (4) if the website has e-journals and provides the function of categorization and searching, and the number of the e-journals is over 10, the value is 1
The resource quality of information service		
2.1.1	Readability	According to whether the contents on the hospital website are easy to be understood by users, readability has four levels valued, respectively, 0, 0.34, 0.67 and 1.
2.1.2	Accuracy	The accuracy concerns with the accuracy of information published on the website, pronunciation and grammar. According to this accuracy, the item has four levels valued, respectively, 0, 0.34, 0.67 and 1.
2.1.3	Completeness	The completeness is whether the information released by the website covers a sufficiently wide range of topics. According to this completeness, the item has four levels valued, respectively, 0, 0.34, 0.67 and 1.
2.1.4	Information source	The information source has five sub-items: (source of the article, reprint mark, reprint review, online reference links and reminding.) it has two levels: “Established” valued 1 and “to be established” valued 0. The total score is 3. The calculation of the value of the quantitative standard is: 1/3(min(3, ∑_*i*=1_^*n*^*W*_*i*_)) “*n*” is the number of the sub-items and equals 5. *W_i_* is the specific value of the *i* sub-item. The “min” function is used to find the smallest value in the given parameter list.
Information service resources management		
2.2.1	Copyright	The copyright has two levels: “Established” valued 1 and “to be established” valued 0.
2.2.2	Qualification licenses for website operation	The qualification licenses for website operation includes ten sub-items: (internet drug information service license, MOH health information service network management review, internet medical and health information service license, internet education information service license, internet domain name accreditation, internet publishing license, internet broadcasting program license, internet payment license, value added telecommunication business license and others.) it has two levels: “Established” valued 1 and “to be established” valued 0. The total score is 5. The calculation of the value of the quantitative standard is: 1/5(min(5, ∑_*i*=1_^*n*^*W*_*i*_)) “*n*” is the number of the sub-items and equals 10. *W_i_* is the specific value of the i sub-item. The “min” function is used to find the smallest value in the given parameter list.
2.2.3	Author's identity	The Author's identity includes six sub-items: (name, education, title, organization, contact and others.) it has two levels: “Established” valued 1 and “to be established” valued 0. The total score is 4. The calculation of the value of the quantitative standard is: 1/4(min(4, ∑_*i*=1_^*n*^*W*_*i*_)) “*n*” is the number of the sub-items and equals 6. *Wi* is the specific value of the i sub-item. The “min” function is used to find the smallest value in the given parameter list.
2.2.4	Title marking	The title marking item has four levels: (1) if the website does not mark any titles, the value is 0 (2) if the homepage or a certain web page has a marked title, the value is 0.34 (3) if the homepage and other pages all have marked titles, the value is 0.67 (4) if the homepage and other pages have different title marks, the value is 1 The level of the sub-item is figured out through the random examination of 20 page titles of the hospital's website.
2.2.5	Accessibility of resources	According to the number of the website's invalid links, the accessibility of resources has four levels: (1) if the number of invalid links is no less than 10 (10 included), the value is 0 (2) if the number of invalid links is between 5 and 10, the value is 0.34 (3) if the number of invalid links is less than 5 (5 included), the value is 0.67 (4) if the website has no invalid links, the value is 1 The number of invalid links is figured out through the random examination of 20 internal links of the website.
2.2.6	Release date	The release date has four levels: (1) if the publishing time or date is not marked, the value is 0 (2) if the time or date is marked, the value is 0.5 (3) if the time and date are both marked, the value is 1
Information service technology		
2.3.1	Information classification	The information classification includes five sub-items: (1) the layout of pages on the website (2) the homepage has information classification (3) the information classification is reasonable (4) the information classification meets users' demands (5) the names of classified information are easy to understand The above five sub-items have four levels: Excellent, good, general and bad, valued, respectively, 0, 0.34, 0.67 and 1. The calculation of the value of this item is: 0.2 × (*W*_1_ + *W*_2_ + *W*_3_ + *W*_4_ + *W*_5_) W_1_,W_2_,W_3_,W_4_ AndW_5_ are, respectively, the values of the five sub-items.
2.3.2	Information stratification	According to the number of the grades of information classification, the item has four levels: (1) if the number of the grades is no less than 10, the value is 0 (2) if the number of the grades is between 5 and 10, the value is 0.34 (3) if the number of the grades is between 4 (4 included) and 5 (5 included), the value is 0.67 (4) if the number of the grades is less than 4, the value is 1
2.3.3	Navigation mark	The navigation mark includes five items: (A back link to the last page, a back link to the homepage, a back link to the information classification page, a link to homepage prompt message and a navigation map for the website.) it has two levels: “Established” valued 1 and “to be established” valued 0. The total score is 3. The calculation of the value of the quantitative standard is: 1/3(min(3, ∑_*i*=1_^*n*^*W*_*i*_)) “*n*” is the number of the sub-items and equals 5. W*_i_* is the specific value of the *i* sub-item. The level of the sub-item is figured out through the random examination of 20 page titles of the hospital's website. The “min” function is used to find the smallest value in the given parameter list.
2.3.4	Format specification	The format specification includes eight sub-items: (well-designed pages, user-friendly font size, uniform page layout, appropriate page margin, pagination on long pages, a horizontal scroll bar on large pages, zoom-in and zoom-out function, all browsers supported.) it has two levels: “Established” valued 1 and “to be established” valued 0. The total score is 7. The calculation of the value of the quantitative standard is: $\frac{1}{4}\left(\min\left(4,\overset{\text{n}}{\underset{i=1}{\sum }}{\text{w}}_{i}\right)\right)$ “n” is the number of the sub-items and equals 8. Wi is the specific value of the i sub-item. The level of the sub-items is figured out through the random examination of 20 pages of the hospital's website. The “min” function is used to find the smallest value in the given parameter list.
2.3.5	Specification of domain name	The specification of domain name is whether the URL of the hospital website is composed of the hospital's English name or Chinese pinyin initials, whether it has multiple corresponding domain names and whether users find it easy to remember and use. This item has three levels: (1) if the domain name is not composed of the hospital's English name or Chinese pinyin initials, and it does not have multiple corresponding domain names, the value is 0 (2) if the domain name is composed of the hospital's English name or Chinese pinyin initials, while it does not have multiple corresponding domain names, the value is 0.5 (3) if the domain name is composed of the hospital's English name or Chinese pinyin initials, and it has multiple corresponding domain names formed by the hospital's English name and Chinese pinyin initials, the value is 1
2.3.6	Language	If the website only has simple Chinese version, the value is 0.5; if it has two or more language versions including Chinese, the value is 1.
Information architecture		
2.4.1	Time taken to open the homepage	According to the time users need to open the hospital website's homepage, the time taken to open the homepage (before flashes and videos are played) has four levels: (1) if the homepage open time exceeds 10 seconds, the value is 0 (2) if the homepage open time is between 8 and 10 (10 included) seconds, the value is 0.34 (3) if the homepage open time is between 4 and 8 (8 included) seconds, the value is 0.67 (4) if the homepage open time is no more than 4 seconds, the value is 1
2.4.2	Digital multimedia function	The digital multimedia function refers to dynamic forms of information services provided by the hospital website, including pictures, sounds, videos, flashes, dynamic pages and 3D interaction programs. Each of the above six sub-items has two levels: “Established” valued 1 and “to be established” valued 0. The total score is 4. In case that advertisement appears in the forms of dialog boxes and float bars on the website, 1 point is deducted. The calculation of the value of the quantitative standard is: $\frac{1}{4}\left(\min\left(4,\overset{\text{n}}{\underset{i=1}{\sum }}{\text{w}}_{i}\right)-R\right)$ “*n*” is the number of the sub-items and equals 6. W*_i_* is the specific value of the *i* sub-item. R is the case that incurs any point deduction. The level of the sub-items is figured out through the random examination of 20 pages of the hospital's website. The “min” function is used to find the smallest value in the given parameter list.
2.4.3	Website Pageview	The website page view is the function to calculate the number of visitors to the hospital website. According to its function, the item has two levels: “Established” valued 1 and “to be established” valued 0.
2.4.4	Background data	The background data refers to that the information service resources on the website are posted in the format and form of background data. It has four sub-items: (1) if the resources are not in the format and form of background data, the value is 0 (2) if part of the contents on the website is in the format and form of background data, the value is 0.34 (3) if half or more of the contents of the website are in the format and form of background data, the value is 0.67 (4) if all the contents of the website are in the format and form of background data, the value is 1

Source: the author.

**Table 3:** Comprehensive Evaluations of the Information Service of a Hospital Information Platform's Information Platform.

No.	Period	Date	1.1	1.2	1.3	1.4	2.1	2.2	2.3	2.4	Total	Total score
1	Pre-revision	2016/12/20	0.088	0.000	0.000	0.003	0.033	0.025	0.021	0.025	0.194	19.4
2	Pre-revision	2017/01/05	0.088	0.000	0.000	0.003	0.042	0.025	0.021	0.025	0.204	20.4
3	Post-revision	2017/01/20	0.151	0.205	0.086	0.051	0.096	0.071	0.033	0.043	0.735	73.5
4	Post-revision	2017/02/06	0.151	0.205	0.089	0.051	0.101	0.071	0.033	0.043	0.744	74.4
5	Post-revision	2017/08/18	0.157	0.205	0.089	0.055	0.113	0.078	0.037	0.043	0.777	77.7
6	Post-revision	2018/02/06	0.157	0.205	0.089	0.055	0.113	0.078	0.037	0.038	0.773	77.3
7	Post-revision	2018/08/03	0.157	0.205	0.089	0.055	0.113	0.078	0.037	0.041	0.775	77.5
8	Post-revision	2019/02/21	0.157	0.205	0.090	0.058	0.116	0.090	0.037	0.041	0.794	79.4
9	Post-revision	2019/08/21	0.161	0.205	0.101	0.067	0.116	0.090	0.037	0.041	0.817	81.7
10	Post-revision	2020/01/09	0.166	0.205	0.117	0.067	0.116	0.090	0.038	0.045	0.843	84.3

**Table 4:** Evaluation score of the information services of a Hospital Information Platform's website in China of Growth of rate in empirical research.

Date	1	2	1.1	1.2	1.3	1.4	2.1	2.2	2.3	2.4
2016/12/20	0	0	0	0	0	0	0	0	0	0
2017/1/5	0.5000	1.0777	1	0	0	1	1.2923	1	1.0183	1
2017/1/20	4.6790	2.1007	1.7159	0	0	17.0000	2.2857	2.8400	1.5569	1.7200
2017/2/6	1.0088	1.0169	1	1	1.0351	1	1.0521	1	1.0154	1
2017/8/18	1.0313	1.0843	1.0389	0.9988	1.0055	1.0819	1.1213	1.0990	1.1229	0.9942
2018/2/6	1.0013	0.9733	1	1	1.0052	1	1	1	0.9975	0.8958
2018/8/3	1	1.0147	1	1	1	1	1	1	1	1.0588
2019/2/21	1.0144	1.0449	1	1	1.0103	1.0471	1.0262	1.1534	1	1
2019/8/21	1.0744	1	1.0250	1	1.1140	1.1586	1	1	1	1
2020/1/9	1.0473	1.0337	1.0305	1	1.1588	1	1	1	1.0251	1.1099

## Data Availability

The Survey results and network traffic data used to support the findings of this study are available from the corresponding author upon request.
